# The "Times" on Lead Poisoning

**Published:** 1898-10-15

**Authors:** 


### THE "TIMES" ON LEAD POISONING.

It seems a pity that the special correspondent of the *Times* who is at present writing a series of articles on "Lead Poisoning in the Potteries" should go out of his way, as he does, to insult the medical men practising in that district by his comments on their certificates. He says: "It seems to be a settled thing in many quarters that factory hands not only must have lead poisoning, but may not have anything else. . . . The doctors have lead upon the brain as well as the patients, and the craze is carried so far as to make the greatest caution necessary in accepting alleged cases, especially if they are of a startling kind." All this merely shows bias, and tends to throw doubt upon the reliability of all else that he brings forward, for it must be remembered that these certificates are given by responsible medical men who are liable to heavy penalties for any false statements they may contain, and thus, as evidence, are of far more value than the "facts" collected by any irresponsible correspondent, however influential may be the newspaper under whose ægis he writes. For ourselves, we prefer to trust to the opinion of the Chief Inspector of Factories, who considers that these certificates understate rather than overstate the case, and says that "there is good reason to fear that the numbers would be much larger if all the cases were known."

The *Times* reporter is, however, on surer ground when he comes to deal with the comparative efficiency of the various causes which go to produce lead poisoning, and he is no doubt quite correct, indeed he is but following a well worn track, in laying a large proportion of the blame upon idiosyncrasy and upon the careless habits of the workers. All investigators into this subject have noticed the extraordinary immunity to the evil effects of metallic poisons which certain workers among them exhibit. Not only in the potteries is this the case, and we may add not only in regard to lead. Workers in arsenical dyes, in phosphorus, in Scheele's green, and many other poisonous compounds, exhibit the same peculiarity. In all of them we may find individuals who work without suffering in the midst of surroundings which are apparently destructive, and destructive quickly to their neighbours, and we can hardly escape the conclusion that there is some personal idiosyncrasy at work, causing the poison sometimes to be effective and sometimes to be inert.

In regard to many of these trades we know of what this personal peculiarity consists. Among phosphorus workers a carious tooth becomes the point of entry by means of which the poison attacks its victim; among those who work in the midst of dust highly charged with arsenical compounds, moisture of skin, and perhaps some peculiarity in the character of the perspiration in certain regions of the body becomes a predisposing influence which determines whether an individual shall suffer or remain immune; and very often, no doubt, among workers in lead, the efficacy or otherwise of the kidneys decides whether the minimum quantity of lead which must in every case be absorbed shall be excreted or shall accumulate. But overshadowing all these factors in the production of lead poisoning is the individual care each worker takes of his own person. All this is perfectly well known to all who have investigated the matter. Still it is well to have the state of affairs put plainly before the public. "In every single factory I went into," says the *Times* correspondent, "I found men and women who had worked in the lead for very long periods—ten, twenty, forty, and even fifty years—without a trace of ill-effects. . . . Directly I asked the question as to their own health they would volunteer the explanation, 'You see, sir, it depends on a man himself.' These immune workers do not always keep the rules. I met with several who flatly refused to wear overalls, for instance, but they are cleanly in their person."

This is an aspect of the question on which it is quite right to insist; at the same time we may fairly doubt whether it is either wise or even correct to say that the state of the workshop "is the least important of all the conditions" which promote lead poisoning. We must never forget that all this necessary cleaning of the person is a great labour added to the day's work, and that among the most important measures which can be insisted on for the protection of the average worker are the provision of mechanical ventilation so as to remove poisonous dust with all speed from the workshops and thus to diminish the amount of cleansing necessary, and the provision of ample and appropriate lavatory arrangements, so as to render the cleansing process itself as little irksome as may be. There are few points to which women inspectors in these trades can more usefully turn their attention than to the facilities provided for cleansing the person not merely with ease but with decency, for there is some reason to believe that part at least of the special liability of young women to lead poisoning is due to feelings of modesty which should be considered creditable to them.

